# Optimizing dengue vaccination strategies

**DOI:** 10.1371/journal.pmed.1005068

**Published:** 2026-04-27

**Authors:** Annelies Wilder-Smith

**Affiliations:** 1 Department for Immunization and Vaccines, World Health Organization, Geneva, Switzerland; 2 Heidelberg Institute of Global Health, University of Heidelberg, Heidelberg, Germany

## Abstract

Dengue is a rapidly expanding global health threat driven by urbanization, climate change, and complex transmission dynamics. In this Perspective, Annelies Wilder-Smith details why modeling and tailored vaccination programs will be critical to developing effective, context-specific strategies to reducing disease burden.

## Teaser

Dengue is a rapidly expanding global health threat driven by urbanization, climate change, and complex transmission dynamics. Modeling and tailored vaccination programs will be critical to developing effective, context-specific strategies to reducing disease burden.

Dengue virus (DENV), comprising four antigenically distinct serotypes (DENV-1–4), remains a major public health challenge in tropical and subtropical regions regions where the vector—primarily *Aedes* mosquitoes—exists [1]. Outbreaks are becoming more frequent and severe, with the disease spreading into more temperate regions, including Southern Europe and parts of the southern United States. Dengue is also increasingly affecting international travelers [2]. While primary infections are often asymptomatic or mild, secondary infections with a different serotype carry a higher risk of severe disease due to antibody-dependent enhancement [3]. Severe dengue is characterized by capillary leakage and thrombocytopenia, with the potential to progress to shock syndrome [1,3]. Because it is difficult to predict which patients will develop severe disease, many individuals with dengue are hospitalized for close monitoring and supportive care, placing a substantial burden on already strained health systems.

Poor urban planning creates ideal breeding conditions for *Aedes* mosquitoes through inadequate water storage practices, ineffective waste management, and poor drainage, all of which generate standing water. These factors, combined with high population density and favorable climatic conditions such as warmth and humidity, facilitate mosquito proliferation and virus transmission. Human mobility, population immunity, and the strength of public health systems further shape transmission dynamics [1]. With rapid urbanization and climate change, dengue incidence is poised to increase further, underscoring that vector control alone is insufficient and that additional tools, including vaccination, are needed.

CYD-TDV (Dengvaxia) was the first dengue vaccine to be licensed; however, its programmatic rollout was hampered by the requirement for pre-vaccination screening, as only seropositive individuals were eligible to receive the vaccine, leading to significant implementation complexities [4]. TAK-003 (Qdenga) is the second licensed dengue vaccine and does not require pre-vaccination screening [5]. It is administered as a two-dose series, given three months apart. As is typical for live attenuated dengue vaccines, its efficacy is higher in individuals with prior dengue exposure (seropositive) than in those who are seronegative. The vaccine provides the strongest protection against DENV-2, likely reflecting its DENV-2 backbone, but shows no efficacy against DENV-3 in seronegative individuals [6–8].

Based on these characteristics, the World Health Organization (WHO) recommends its use primarily in high-transmission settings, defined by ≥60% seroprevalence by age nine, with vaccination targeted at children aged 6–16 years as under such settings the public health impact is greatest [5].

Given the heterogeneity of dengue epidemiology across settings—shaped by demographic, socioeconomic, climatic, and geographic factors—vaccination strategies must be tailored to country-specific contexts. Mathematical modeling is therefore helpful to inform optimal introduction strategies. In Thailand, where the above WHO criteria for vaccine introduction are met due to high transmission intensity and seroprevalence, a dynamic transmission model incorporating all four serotypes, seasonality, and baseline serostatus was used to evaluate multiple vaccination strategies for TAK-003 [9]. These included routine childhood vaccination, combinations with catch-up campaigns, and integration with existing school-based programmes such as HPV vaccination. Across all scenarios, vaccination substantially reduced symptomatic and severe dengue. Across a range of vaccination strategies evaluated, TAK-003 would prevent 41%−57% of dengue cases and 47%−70% of hospitalizations over a 20-year period. Among these, routine vaccination of children aged 6 years, combined with 10 additional catch-up cohorts, was identified as the most cost-effective approach, with projected savings of US$1,786 million, at an assumed price of $30 per dose [9]. A more pragmatic strategy—introducing TAK-003 into the Thai immunization programme through co-administration with the existing human papillomavirus vaccination at age 11—would still yield substantial benefits. This approach is estimated to prevent 44% of cases and 53% of hospitalizations, while generating savings in Thailand of US$1,346 million over 20 years.

In contrast to Thailand, Singapore represents a lower-transmission setting with seroprevalence below WHO threshold and a different age distribution of disease burden, characterized by higher incidence in young adults and more severe outcomes in older individuals. Modeling tailored to Singapore’s epidemiology suggests that vaccinating individuals aged 17–30 years would maximize case reduction, while targeting those aged 51–70 years would yield the greatest reduction in hospitalizations [10]. These findings highlight the importance of adapting global recommendations to local transmission patterns.

Brazil was the first country to introduce TAK-003 programmatically. Initial real-world data from its use among adolescents during the 2024 dengue outbreak in the state of São Paulo showed an adjusted vaccine effectiveness of 50% after the first dose and 62% after the second dose against symptomatic dengue, consistent with findings from Phase 3 trials [11]. Cost-effectiveness analyses for the Brazilian setting are still relatively limited. First experiences with TAK-003 have also been reported in travelers [12], but no benefit-risk assessments or vaccine effectiveness data are available for this group.

Given its high dengue burden, Brazil has also licensed a third dengue vaccine, Butantan-DV, a single-dose candidate evaluated in a large five-year trial conducted in the country [13]. At present, this vaccine is available only in Brazil and is not accessible to the global market due to marketing agreements with Merck. Meanwhile, Merck and other independent vaccine developers in India are conducting Phase 3 trials of dengue vaccines based on the same virus backbone as Butantan-DV; however, global licensure is still expected to take several years.

Dengue-endemic countries should carefully consider how best to deploy TAK-003, currently the only available dengue vaccine globally. When implemented through strategies aligned with local epidemiology and health system priorities, TAK-003 has the potential to substantially reduce dengue burden. Modeled outcomes consistently show that higher vaccine coverage leads to greater reductions in disease, underscoring the importance of achieving high uptake. Future research should focus on real-world effectiveness, particularly in seronegative populations, as well as the durability of protection. As more countries consider vaccine introduction, flexible, data-driven modeling approaches will be essential to guide context-specific decision-making.

## Abbreviations

DENV: dengue virus
WHO: World Health Organization
